# Hyperuricemia as an independent risk factor for achilles tendon rupture in male: a case–control study

**DOI:** 10.1186/s13018-024-04698-9

**Published:** 2024-04-01

**Authors:** Dongliang Chen, Jinwei Liu, Zhaohui Zhu, Zengfang Zhang, Deheng Liu, Liangxiao Zheng

**Affiliations:** Department of Hand and Foot Surgery, Orthopedic Center, Qilu Hospital (Qingdao), Cheeloo College of Medicine, Shandong University, Qingdao, China

**Keywords:** Achilles tendon rupture, Hyperuricemia, Risk factor, Receiver operating characteristic

## Abstract

**Objective:**

To study the correlation between achilles tendon rupture (ATR) and hyperuricemia, also verify the known risk factors for ATR.

**Methods:**

A retrospective review of 488 subjects was performed (182 with Achilles tendon rupture, 306 controls with ankle sprains). Demographic variables and risk factors for rupture were tabulated and compared. The baseline data and related indicators were compared, and the risk factors of ATR were analyzed by constructing a binary logistic regression model.

**Results:**

Univariate logistic analysis showed that BMI, smoking, and hyperuricemia were risk factors for the development of ATR (OR = 1.65, 95%CI 1.13–2.42, *P* = 0.01; OR = 1.47, 95%CI 1.00–2.24, *P* < 0.05; OR = 2.85, 95%CI 1.84–4.42, *P* < 0.01). Multifactorial analysis showed that BMI ≥ 25 kg/m^2^, smoking, and hyperuricemia were independent risk factors for the development of ATR (OR = 1.66, 95%CI 1.11–2.49, *P* = 0.01; OR = 2.15, 95%CI 1.28–3.60, *P* < 0.01; OR = 3.06, 95%CI 1.92–4.89, *P* < 0.01). Among the blood biochemical indicators, total cholesterol (TC) and uric acid (UA) were independent risk factors for the occurrence of ATR (OR = 1.54, 95% CI 1.12–2.12, *P* = 0.01; OR = 1.01, 95% CI 1.01–1.01, *P* < 0.01).

**Conclusion:**

Our study confirmed that, as in previous results, higher BMI, smoking, and total cholesterol are risk factors for ATR, Hyperuricemia may contribute to the development of ATR, and adjunctive tests for TC and UA in the blood biochemistry may be helpful in predicting the risk of ATR.

## Introduction

Achilles tendon ruptures (ATR) are a common injury associated with exercise, with an incidence ranging from 5 to 50 cases per 100,000 person-years and appearing to be on the rise in recent decades [1–3]. Achilles tendon rupture can severely impair mobility and affect normal activity and movement [4–6]. In general, this damage is more common in male aged between 40 and 50 years old. In fact, the ratio of male injuries to female injuries ranged between 2:1 and 12:1 [5]. Preventing the occurrence of ATR becomes an important option to reduce the health risks in this population.

Several risk factors for ATR have been identified in previous studies, including age, sex, body mass index (BMI), race, smoking status, use of fluoroquinolones, topical and oral corticosteroids, previous achilles tendinopathy, blood type, and intensity of participation in competitive sports [7–13]. Hyperuricemia is defined as a serum uric acid level greater than 6.8 mg/dL (0.40 μmol/L), and less than 20% of patients with hyperuricemia are estimated to develop gout attacks [14]. Hyperuricemia can cause cellular and metabolic distress [15], leading to degeneration of the tendon extracellular matrix and subclinical inflammation. Interestingly, subclinical tendon inflammation and structural damage have been observed in patients with asymptomatic hyperuricemia [16], with previous literature reporting patellar tendon intimal lesions in 12% of hyperuricemia patients and achilles tendinopathy in 15% of hyperuricemic patients, compared to only 1.9% of normouricemic subjects. Based on the hypothesis that ATR is the result of acute trauma to a chronically degenerative tendon [17]. ATR is more prevalent in patients with hyperuricemia than people with normal uric acid, we thus hypothesize that there may be a potential correlation between ATR and hyperuricemia.

The objective of this study was to investigate the relationship between ATR and hyperuricemia in male and to explore the risk factors associated with the development of ATR, while verifying some previous risk factors. Provide valuable information for clinical study of ATR and its preventive measures.

## Materials and methods

### Subjects

This retrospective cross-sectional cohort study included 182 male patients diagnosed with achilles tendon rupture (ATR) and a control group of 306 male patients with ankle fractures, who were seen at the Qilu Hospital (Qingdao), Cheeloo College of Medicine, Shandong University, between January 2014 and July 2022. Inclusion criteria for the ATR group were: (1) 18 to 70-year-old male patients with ATR and complete clinical data, without other coexisting injuries. Exclusion criteria were: (1) insufficient data to calculate body mass index (BMI) or unclear epidemiological data; (2) open Achilles tendon rupture; (3) systemic or local use of steroids or quinolones. The control group selected patients with ankle injury admitted during the same period, we chosen ankle sprains as the control group because the physicians participating in this study easily identified such patients due to the close site of injury. Furthermore, ankle sprains occur in all ages and are therefore considered a reasonable representative of the general population. Exclusion criteria: There was no previous history of acute ATR, and the same exclusion criteria were used as in the ATR group.

### Variables and data sources

Data for the study were collected using a questionnaire that included variables such as demographic data (age and gender), harmful habits (smoking and drinking), use of uricotelic drugs, past medical history, family medical history, medical conditions, and medication. Additionally, information on endocrine and cardiovascular diseases, as well as specific diseases such as diabetes mellitus, hypertension, and coronary heart disease, were recorded.

### Detection methods

Blood specials were collected from patients after at least 10 h of rest and fasting in the morning. Measurements of triglyceride (TG), total cholesterol (TC), low-density lipoprotein (LDL-C), high-density lipoprotein (HDL-C), glucose (GLU) and lipoprotein levels were performed using a fully automated biochemical analyzer (Modular 7600, Hitachi, Tokyo, Japan). Furthermore, the levels of high-density lipoprotein (HDL-C), apolipoproteins A1 (APO-A1), apolipoprotein B (APO-B), and uric acid (UA) were also measured.

### Statistical analysis

A descriptive analysis was conducted on all variables. IBM SPSS Statistics (version 26, R26.0.0.2, IBM, Chicago, USA) was utilized for statistical analysis. Continuous variables with normal distribution were represented as mean ± standard deviation and analyzed using t-tests or one-way ANOVA. Categorical variables were presented as frequencies [n (%)] and analyzed using the χ^2^ test to determine differences between the two groups. Logistic regression was used to analyze risk factors for ATR, with adjustment made for other confounding factors. Sample size was calculated to determine the number of subjects required to demonstrate equivalence of mean UA in ATR and control cohorts using the two one-sided t-test (TOST) method for equivalence testing, with a = 0.05 and b = 0.20. With data obtained from 182 ATR patients, sample size analysis indicated that 306 controls would achieve 80% power. All assays were two-tailed, and statistical significance was indicated by *P* < 0.05.

## Results

### Clinical information

The clinical data of ATR patients and control subjects are shown in Table 1. The results of the univariate analysis showed that among the included subjects, the proportions of BMI, smokers, and hyperuricemia patients in ATR patients were significantly higher than those in the control group, and the differences were statistically significant (*P* < 0.05). There was no significant difference in the proportion of patients with age, hypertension, coronary heart disease, drinking and hypertension between ATR group and the control group (*P* > 0.05).

**Table 1:** Comparison of clinical data of ATR patients and control group

	ATR (n = 182)	Control (n = 306)	χ^2^	*P* value
Age (year)✭	39.58 ± 9.69	41.79 ± 13.98		0.06
BMI (kg/m2)				
< 25	61(33.52%)	139(45.42%)	6.691	0.010
≥ 25	121(66.48%)	167(54.58%)		
Smoking✮				
No	107(58.79%)	207(67.65%)	3.901	0.048
Yes	75(41.21%)	99(32.35%)		
Drinking✭				
No	161(88.46%)	271(88.56%)	0.001	0.973
Yes	21(11.54%)	35(11.44%)		
Hypertension				
No	150(82.42%)	244(79.74%)	0.527	0.468
Yes	32(17.58%)	62(20.26%)		
Diabetes				
No	179(98.35%)	299(97.71%)	0.232	0.630
Yes	3(1.65%)	7(2.29%)		
Coronary heart disease				
No	178(97.80%)	298(97.38%)	0.083	0.774
Yes	4(2.20%)	8(2.62%)		
Hyperuricemia*				
No	121(66.48%)	260(84.97%)	22.776	< 0.0001
Yes	61(33.52%)	46(15.03%)		

^*^Hyperuricemia: UA≧420 mmol/L ✮Age, unpaired t-test (SPSS). ✮Smoking, 10 cigarettes/day or more. ✮Drinking, 4 bottles of beer(1300 ml)/day, or 2 taels liquor/day

### Analysis of risk factors for the occurrence of ATR

Univariate logistic regression and multivariate logistic regression were used to analyze the risk factors for the occurrence of ATR, and the results were shown in Tables 2 and 3. Univariate logistic analysis showed that BMI, smoking, and hyperuricemia were risk factors for the developmalet of ATR (OR = 1.65, 95%CI 1.13–2.42, *P* = 0.01; OR = 1.47, 95%CI 1.00–2.24, *P* < 0.05; OR = 2.85, 95%CI. 1.84–4.42, *P* < 0.01). Multifactorial analysis showed that BMI ≥ 25 kg/m^2^, smoking and hyperuricemia were independent risk factors for the developmalet of ATR (OR = 1.66, 95%CI 1.11–2.49, *P* = 0.01; OR = 2.15, 95%CI 1.28–3.60, *P* < 0.01; OR = 3.06, 95%CI 1.92–4.89, *P* < 0.01).

**Table 2:** Univariate logistic regression analysis of risk factors for ATR occurrence

	B	S.E	Wald	df	Sig	Exp (B)	95% C.I.for EXP (B)	
							Lower	Upper
BMI	0.50	0.20	6.64	1	0.01	1.65	1.13	2.42
Smoking	0.38	0.19	3.89	1	0.05	1.47	1.00	2.14
Drinking	0.01	0.29	0.00	1	0.97	1.01	0.57	1.80
Hypertension	− 0.18	0.24	0.53	1	0.47	0.84	0.52	1.35
Diabetes	− 0.33	0.70	0.23	1	0.63	0.72	0.18	2.80
Coronary heart disease	− 0.18	0.62	0.08	1	0.77	0.84	0.25	2.82
Hyperuricemia	1.05	0.22	21.82	1	< 0.01	2.85	1.84	4.42

**Table 3:** Multivariate logistic regression analysis of risk factors for ATR occurrence

	B	S.E	Wald	df	Sig	Exp (B)	95% C.I.for EXP (B)	
							Lower	Upper
BMI ≥ 25 kg/m^2^	0.51	0.21	6.11	1	0.01	1.66	1.11	2.49
Smoking	0.76	0.26	8.41	1	< 0.01	2.15	1.28	3.60
Drinking	0.33	0.48	0.47	1	0.49	1.39	0.54	3.53
Hypertension	− 0.71	0.44	2.63	1	0.11	0.49	0.21	1.16
Diabetes	− 0.09	1.08	0.01	1	0.94	0.92	0.11	7.55
Coronary heart disease	0.06	0.95	0.00	1	0.95	1.06	0.16	6.79
Hyperuricemia	1.12	0.24	22.11	1	< 0.01	3.06	1.92	4.89

World Health Organization, BMI, Body mass index. BMI ≥ 25 kg/m^2^ = Overweight

### Correlation between blood biochemical levels and ATR

We analyzed the correlation between blood biochemical parameters and the occurrence of ATR. The result of univariate logistic regression analysis showed that TG, TC, LDL-C, UA, GLU, APO-A1, APO-B were risk factors for the occurrence of ATR (*P* < 0.05, Table 4). The result of multivariate logistic regression showed that TC and UA were independent risk factors for the occurrence of ATR (OR = 1.54, 95% CI 1.12–2.12, *P* = 0.01; OR = 1.01, 95% CI 1.01–1.01, *P* < 0.01) (Table 5).

**Table 4:** Univariate analysis of the correlation between blood biochemical levels and ATR

	B	S.E	Wald	df	Sig	Exp (B)	95% C.I.for EXP (B)	
							Lower	Upper
TG	0.25	0.09	8.46	1	< 0.01	1.29	1.09	1.52
TC	0.52	0.11	20.73	1	< 0.01	1.67	1.34	2.09
HDL-C	0.31	0.36	0.75	1	0.39	1.37	0.67	2.79
LDL-C	0.29	0.12	5.58	1	0.02	1.33	1.05	1.69
UA	0.01	0.00	38.94	1	< 0.01	1.01	1.01	1.01
GLU	− 0.18	0.09	4.25	1	0.04	0.84	0.71	0.99
APO-A1	0.83	0.34	5.86	1	0.02	2.29	1.17	4.47
APO-B	0.80	0.38	4.38	1	0.04	2.22	1.05	4.70

TG, Triglyceride. TC, Total cholesterol. HDL-C, High-density lipoprotein. LDL-C, Low-density lipoprotein. UA, Uric acid. GLU, Glucose. APO-A1, Apolipoproteins A1. APO-B, Apolipoprotein B

**Table 5:** Multivariate analysis of the correlation between blood biochemical levels and ATR

	B	S.E	Wald	df	Sig	Exp (B)	95% C.I.for EXP (B)	
							Lower	Upper
TG	0.13	0.09	2.17	1	0.14	1.14	0.96	1.35
TC	0.43	0.16	6.94	1	0.01	1.54	1.12	2.12
HDL-C	− 0.34	0.48	0.49	1	0.49	0.71	0.28	1.84
LDL-C	0.24	0.14	3.08	1	0.08	1.27	0.97	1.65
UA	0.01	0.00	32.82	1	< 0.01	1.01	1.01	1.01
GLU	− 0.18	0.10	3.40	1	0.07	0.84	0.70	1.01
APO-A1	0.61	0.44	1.92	1	0.17	1.85	0.78	4.40
APO-B	− 0.04	0.53	0.01	1	0.94	0.96	0.34	2.73

TG, triglyceride. TC, total cholesterol. HDL-C, high-density lipoprotein. LDL-C, low-density lipoprotein. UA, uric acid. GLU, glucose. APO-A1, apolipoproteins A1. APO-B, apolipoprotein B

**Fig. 1:**
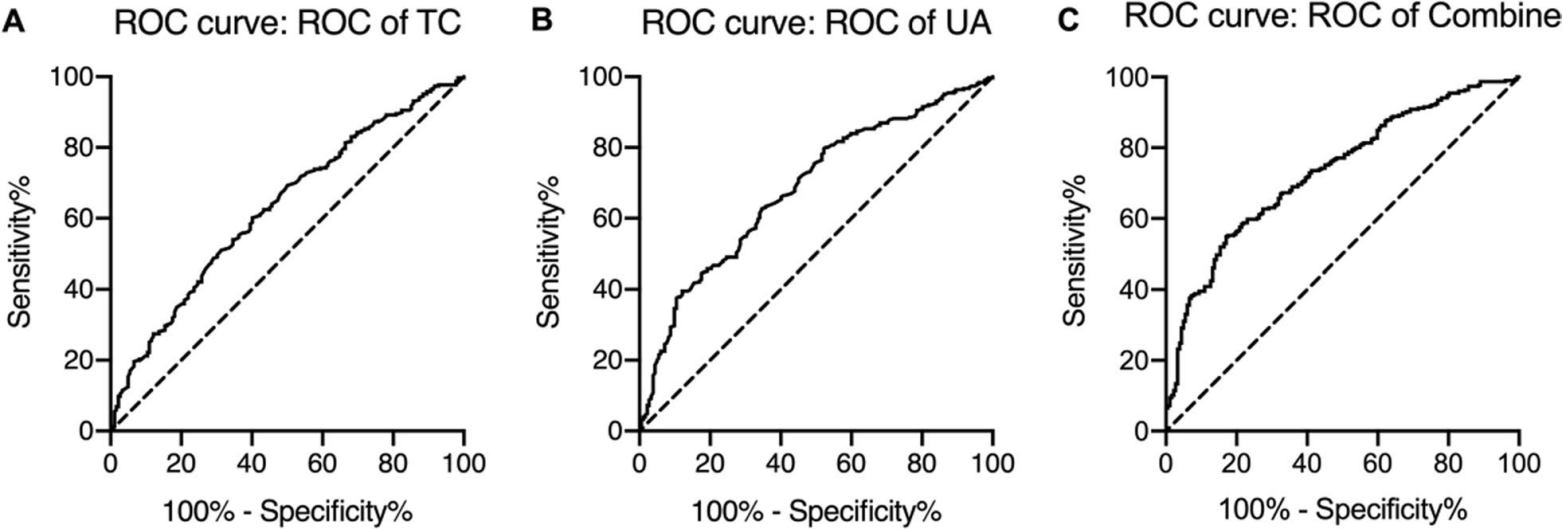
The receiver operating characteristic (ROC) of TC, UA and the combination of the them for the diagnosis of ATR. TC, total cholesterol. UA, uric acid

The results of ROC analysis showed that the area under the curve (AUC) for both TC and UA diagnosis alone and in combination was > 0.6, with a higher sensitivity for UA diagnosis alone and a higher specificity for TC diagnosis in combination with UA (Fig. 1, Table 6).

**Table 6:** The result of ROC for blood biochemical levels

	Area	Std. error	95% CI	*P* value	Sensitivity (%)	Specificity (%)
TC	0.63	0.03	0.58–0.68	< 0.01	60.31	59.89
UA	0.68	0.02	0.64–0.73	< 0.01	62.75	65.38
Combine	0.67	0.03	0.62–0.72	< 0.01	54.90	82.97

TC, total cholesterol. UA, Uric acid.

## Discussion

This case control study has found that hyperuricemia is an autonomous risk factor for ATR in the male Chinese population. The analysis of blood biochemical test results showed a considerable difference in uric acid values between the two groups. To date, Studies on the association between hyperuricemia and ATR in male were scarce. Therefore, this research sets an important precedent and plays a crucial role in the prevention and treatment of ATR in exercise active male aged around 40 years old. In order for the conclusions of this study to be considered valid, it is important that the sample demonstrate similarities in risk factors for ATR as compared to previously published studies. This study sample accurately represents the general population of ATR in coastal China, with predominantly male individuals suffering primarily exercise-related injuries. Additionally, TC is a significant risk factor, and other known risk factors, such as smoking, tend to increase the risk of ATR.

Hyperuricemia, a disorder characterized by abnormal purine metabolism, has been found to have an impact on tendons, although its significance in this regard is often underestimated and not fully comprehended [15, 18]. Although evidence linking hyperuricemia to tendinopathy is limited, it is more evident that crystal deposition in and around tendons during gout attacks can cause cell death. Dodds et al. [19] found that 30 patients with ATR had significantly higher uric acid levels when compared to healthy controls. Additionally, Beskin reported a 14% incidence of gout in 42 consecutive patients with ATR [20].The precise mechanism by which Achilles tendon injury occurs remains unclear, although restricted blood supply and degenerative changes are generally believed to be the primary causes [7, 21]. In a retrospective study investigating the relationship between tendon pathologies and uric acid levels, Abate et al. [15] found that elevated serum uric acid levels disrupt proteoglycan metabolism, which is the underlying cause of tendon injury. Recent research evidence suggests that asymptomatic hyperuricemia may be a predisposition of ATR by impeding the normal functions of tendon stem/progenitor cells (TSPCs) [22]. There is also research evidence that MSU crystals directly interact with tenocytes to reduce cell viability and function [23]. Andia I et al. found that urate crystals caused pro-inflammatory response drives the progression of tendinopathy [24]. An MRI study of 45 cases of Achilles tendon rupture by Bäcker HC et al. [25] confirmed that was evidence of diffuse degeneration in each achilles tendon. Achilles tendon degeneration or tendinopathy can lead to the mechanical failure of the achilles tendon. Based on this analysis, it was hypothesized that an increase in uric acid levels caused secondary ATR due to Achilles tendinopathy. However, there is no direct evidence to confirm the causal relationship between the increase in uric acid levels, Achilles tendinopathy and ATR, and further studies are needed to provide more comprehensive data and pathological findings.

Several limitations are associated with this study. Firstly, the retrospective survey design used in this comparative study only provides a relevant basis and cannot confirm the causal relationship, which warrants confirmation through a prospective investigation study conducted on a large sample size of natural populations in the community. Secondly, the relatively small number of ATR cases prohibit subsequent subgroup analysis, particularly with regards to other factors contributing to ATR, such as congenital malformation factors like Haglund malformation that could be associated with ATR. Thirdly, due to the fluctuation of serum uric acid levels and sex differences, we only analyzed admitted fasting blood of male patients, and the lack of follow-up review results also limits the scope of the study. Fourth, the survey area is in the coastal region and may have a higher incidence of hyperuricemia, which is another limitation of this study. Fifth, since the duration of hyperuricemia cannot be determined at the time of diagnosis, there is a lack of data on the duration of hyperuricemia in patients with ATR in this study, and it remains unclear whether patients with longer hyperuricemia duration are more prone to tendon rupture. Sixth, there is an absence of histological data regarding tendon tissue, which will be the main focus of this aspect of the study in future clinical research.

## Conclusion

Our study confirmed that, as in previous results, higher BMI, smoking, and total cholesterol are risk factors for ATR, Hyperuricemia may contribute to the development of ATR, and adjunctive tests for TC and UA in the blood biochemistry may be helpful in predicting the risk of ATR.

## References

[1] Sheth U, Wasserstein D, Jenkinson R, Moineddin R, Kreder H, Jaglal SB (2017). The epidemiology and trends in management of acute Achilles tendon ruptures in Ontario, Canada: a population-based study of 27 607 patients. Bone Jt J.

[2] Ganestam A, Kallemose T, Troelsen A, Barfod KW (2016). Increasing incidence of acute achilles tendon rupture and a noticeable decline in surgical treatment from 1994 to 2013. A nationwide registry study of 33,160 patients. Knee Surg Sports Traumatol Arthrosc.

[3] Egger AC, Berkowitz MJ (2017). Achilles tendon injuries. Curr Rev Musculoskelet Med.

[4] Maffulli N, Peretti GM (2020). Treatment decisions for acute achilles tendon ruptures. Lancet.

[5] Gajhede-Knudsen M, Ekstrand J, Magnusson H, Maffulli N (2013). Recurrence of achilles tendon injuries in elite male football players is more common after early return to play: an 11-year follow-up of the UEFA Champions League injury study. Br J Sports Med.

[6] Maffulli N, Irwin AS, Kenward MG, Smith F, Porter RW (1998). Achilles tendon rupture and sciatica: a possible correlation. Br J Sports Med.

[7] Hess GW (2010). Achilles tendon rupture: a review of etiology, population, anatomy, risk factors, and injury prevention. Foot Ankle Spec.

[8] Alves C, Mendes D, Marques FB (2019). Fluoroquinolones and the risk of tendon injury: a systematic review and meta-analysis. Eur J Clin Pharmacol.

[9] Noback PC, Jang ES, Cuellar DO, Seetharaman M, Malagoli E, Greisberg JK (2017). Risk factors for achilles tendon rupture: a matched case control study. Injury.

[10] Macchi M, Spezia M, Elli S, Schiaffini G, Chisari E (2020). Obesity increases the risk of tendinopathy, tendon tear and rupture, and postoperative complications: a systematic review of clinical studies. Clin Orthop Relat Res.

[11] Yasui Y, Tonogai I, Rosenbaum AJ, Shimozono Y, Kawano H, Kennedy JG (2017). The risk of achilles tendon rupture in the patients with achilles tendinopathy: healthcare database analysis in the United States. Biomed Res Int.

[12] Xergia SA, Tsarbou C, Liveris NI, Hadjithoma M, Tzanetakou IP. Risk factors for achilles tendon rupture: an updated systematic review. Phys Sportsmed. 1–11. 10.1080/00913847.2022.2085505 35670156

[13] Godoy-Santos AL, Bruschini H, Cury J, Srougi M, de Cesar-Netto C, Fonseca LF (2018). Fluoroquinolones and the risk of achilles tendon disorders: update on a neglected complication. Urology.

[14] Campion EW, Glynn RJ, DeLabry LO (1987). Asymptomatic hyperuricemia. Risks and consequences in the normative aging study. Am J Med.

[15] Abate M, Schiavone C, Salini V, Andia I (2013). Occurrence of tendon pathologies in metabolic disorders. Rheumatology (Oxford).

[16] Pineda C, Amezcua-Guerra LM, Solano C, Rodriguez-Henriquez P, Hernandez-Diaz C, Vargas A (2011). Joint and tendon subclinical involvement suggestive of gouty arthritis in asymptomatic hyperuricemia: an ultrasound controlled study. Arthritis Res Ther.

[17] Fedorczyk JM, Barr AE, Rani S, Gao HG, Amin M, Amin S (2010). Exposure-dependent increases in IL-1beta, substance P, CTGF, and tendinosis in flexor digitorum tendons with upper extremity repetitive strain injury. J Orthop Res.

[18] Johnson RJ, Bakris GL, Borghi C, Chonchol MB, Feldman D, Lanaspa MA (2018). Hyperuricemia, acute and chronic kidney disease, hypertension, and cardiovascular disease: report of a scientific workshop organized by the National Kidney Foundation. Am J Kidney Dis.

[19] Dodds WN, Burry HC (1984). The relationship between achilles tendon rupture and serum uric acid level. Injury.

[20] Beskin JL, Sanders RA, Hunter SC, Hughston JC (1987). Surgical repair of achilles tendon ruptures. Am J Sports Med.

[21] Gil-Melgosa L, Grasa J, Urbiola A, Llombart R, Susaeta Ruiz M, Montiel V (2021). Muscular and tendon degeneration after achilles rupture: new insights into future repair strategies. Biomedicines.

[22] Liang J, Chen B, Li Y, Nie D, Lei C, Yang Q (2022). Asymptomatic hyperuricemia is associated with achilles tendon rupture through disrupting the normal functions of tendon stem/progenitor cells. Stem Cells Int.

[23] Chhana A, Callon KE, Dray M, Pool B, Naot D, Gamble GD (2014). Interactions between tenocytes and monosodium urate monohydrate crystals: implications for tendon involvement in gout. Ann Rheum Dis.

[24] Andia I, Abate M (2016). Hyperuricemia in tendons. Adv Exp Med Biol.

[25] Backer HC, Wong TT, Vosseller JT (2019). MRI assessment of degeneration of the tendon in achilles tendon ruptures. Foot Ankle Int.

